# Long-term clinical outcomes of carvedilol versus nebivolol in patients with ST-segment elevation myocardial infarction treated with second-generation drug-eluting stents: insights from a Korean nationwide multicenter prospective registry

**DOI:** 10.3389/fcvm.2026.1865226

**Published:** 2026-08-25

**Authors:** Jihun Ahn, Hye Yon Yu, Seung-Woon Rha, Se Yeon Choi, Jae Kyeong Byun, Soohyung Park, Cheol Ung Choi, Youngkeun Ahn, Dongkyu Jin, Byoung Geol Choi

**Affiliations:** 1 Department of Cardiology, Soonchunhyang University Gumi Hospital, Gumi, Republic of Korea; 2 School of Nursing, College of Medicine, Soonchunhyang University, Asan, Republic of Korea; 3 Cardiovascular Center, Korea University Guro Hospital, Seoul, Republic of Korea; 4 Department of Cardiology, Chonnam National University Hospital, Gwangju, Republic of Korea; 5 Department of Cardiology, Soonchunhyang University Cheonan Hospital, Cheonan, Republic of Korea; 6 Department of Biomedical Laboratory Science, Honam University, Gwangju, Republic of Korea

**Keywords:** carvedilol, drug eluting stent (DES), nebivolol, percutaneous coronary intervention, ST-segment elevation myocardial infarction (STEMI)

## Abstract

**Background:**

Owing to the limited head-to-head comparative evidence between vasodilating *β*-blockers, this study aimed to assess long-term clinical outcomes of nebivolol vs. carvedilol in patients with ST-segment elevation myocardial infarction (STEMI) undergoing percutaneous coronary intervention (PCI) with second-generation drug-eluting stents (DES).

**Methods:**

Data were obtained from the Korea Acute Myocardial Infarction Registry (KAMIR), including the KAMIR–National Institutes of Health (NIH) and KAMIR-V cohorts (2011–2020). Patients with STEMI who underwent successful PCI with second-generation DES and received either carvedilol or nebivolol were retrospectively analyzed. The primary endpoint was 3-year major adverse cardiac events (MACE). Secondary endpoints included individual MACE components, stent thrombosis, and heart failure–related rehospitalization. Inverse probability of treatment weighting (IPTW) was used to adjust for baseline differences.

**Results:**

A total of 6,752 eligible patients with STEMI were identified, baseline differences were effectively minimized using IPTW analysis. Over a median follow-up of 3 years, nebivolol was associated with a higher MACE risk compared with carvedilol (11.1% vs. 9.6%; hazard ratio [HR] 1.176, 95% confidence interval [CI] 1.052–1.314, *p* = 0.005). All-cause mortality (4.4% vs. 3.4%; HR: 1.315, 95% CI: 1.102–1.569, *p* = 0.002) and revascularization (7.0% vs. 6.2%, *p* = 0.043; HR: 1.15, 95% CI: 1.00–1.32, *p* = 0.043) were also higher in the nebivolol group compared with carvedilol.

**Conclusions:**

In this contemporary cohort of patients with STEMI treated with DES, carvedilol was associated with better long-term clinical outcomes, including lower risks of MACE and all-cause mortality, compared with nebivolol.

## 1 Introduction

Beta-blockers remain a cornerstone of secondary prevention after acute myocardial infarction (AMI). Current guidelines recommend long-term beta-blocker therapy as Class I for patients with left ventricular ejection fraction (LVEF) <40% and as Class IIa for those with preserved LVEF, based on landmark randomized trials demonstrating reductions in mortality and recurrent ischemic events (1, 2). Carvedilol, bisoprolol, and metoprolol succinate are recognized as evidence-based beta-blockers with proven benefit in patients with left ventricular systolic dysfunction or prior myocardial infarction (MI) (3–5). However, these pivotal trials were conducted largely before the widespread use of primary percutaneous coronary intervention (PCI) and drug-eluting stents (DES), limiting their applicability to contemporary clinical practice. Nebivolol has emerged as a potential alternative in selected cardiovascular populations. This newer-generation, highly *β*_1_-selective beta-blocker exerts nitric oxide (NO)—mediated vasodilatory effects, improves endothelial function, and favorably influences vascular hemodynamic, suggesting possible advantages in the modern post-MI setting (6). Observational studies have reported improved outcomes with vasodilating beta-blockers compared with conventional agents in patients with MI undergoing PCI (7, 8). Nevertheless, direct comparative data between carvedilol and nebivolol remain limited, and treatment selection in clinical practice is frequently empirical rather than evidence-based. Accordingly, this study aimed to directly compare long-term clinical outcomes between carvedilol, a vasodilating beta-blocker with combined *β*- and *α*_1_-adrenergic receptor blockade (9), and nebivolol, a highly *β*_1_-selective agent with NO-mediated vasodilatory properties, in patients with STEMI underwent primary PCI with second-generation DES.

## 2 Materials and methods

### 2.1 Study design and population

**Figure 1:**
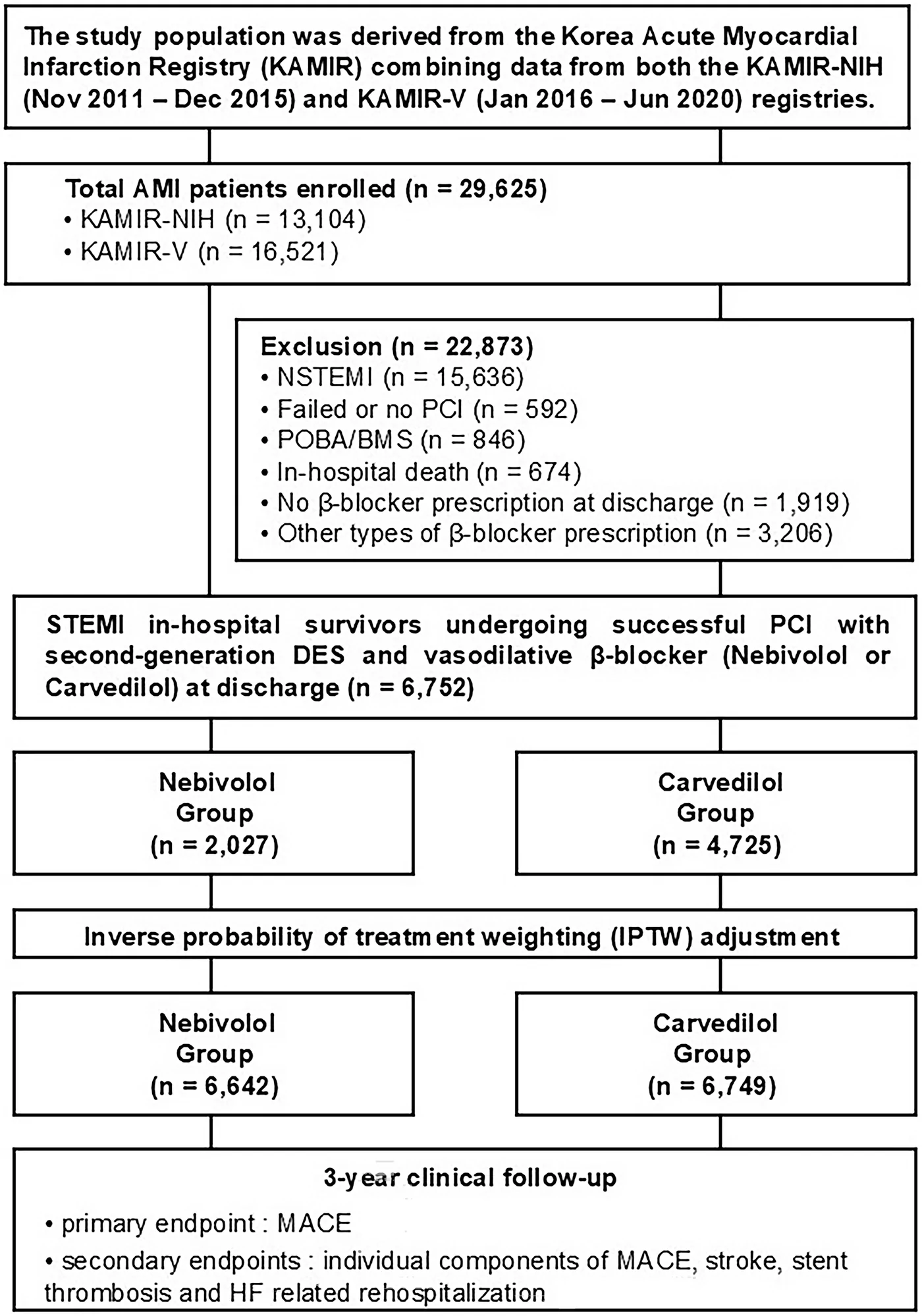
Flow chart.

This retrospective, observational cohort study utilized data from the multicentre Korea Acute Myocardial Infarction Registry (KAMIR), integrating KAMIR–National Institutes of Health (NIH) and KAMIR-V datasets. KAMIR is a nationwide, prospective, multicentre registry capturing real-world data from tertiary hospitals across Korea on AMI management and outcomes (10). Its robust design and systematic follow-up enable longitudinal treatment assessment, providing reliable foundation for comparative effectiveness research. Between November 2011 and June 2020, 29,625 patients with AMI were consecutively enrolled across 20 participating hospitals. Clinical, angiographic, and laboratory data were collected at each participating hospital and entered into the KAMIR database using a secure web-based electronic data capture system. Laboratory measurements, including lipid profiles, were obtained from fasting venous blood samples, typically on the morning following admission, according to the standardized registry protocol. Inclusion criteria were (1) STEMI diagnosis, (2) successful PCI using second generation DES, and (3) carvedilol or nebivolol prescription at discharge. Exclusion criteria included non-ST-segment elevation myocardial infarction (NSTEMI), failed or non-performed PCI, percutaneous old balloon angioplasty (POBA) without stent implantation or other stent types (bare metal stent or first-generation DES), in-hospital death, and absence of beta-blocker therapy or use of beta-blockers other than carvedilol or nebivolol. The final cohort consisted of in-hospital survivors with STEMI treated with either carvedilol or nebivolol (Figure 1).

Study population flow was derived from the Korea Acute Myocardial Infarction Registry (KAMIR), combining KAMIR–NIH (November 2011–December 2015) and KAMIR-V (January 2016–June 2020) data. Among 29,625 patients with acute myocardial infarction, 22,873 were excluded for overlapping reasons, leaving 6,752 in-hospital survivors with ST-segment elevation myocardial infarction (STEMI) who underwent successful PCI using second-generation drug-eluting stents (DES) and were prescribed vasodilative *β*-blockers at discharge. The final cohort consisted of 2,027 patients treated with nebivolol and 4,725 with carvedilol.

*Some patients met multiple exclusion criteria, resulting in category overlap.

### 2.2 Study definitions and clinical endpoints

Successful PCI was defined as restoration of Thrombolysis in Myocardial Infarction (TIMI) flow grade 3 without in-hospital major adverse cardiac events. The primary endpoint was 3-year major adverse cardiac events (MACE), defined as a composite of all-cause death, MI, or any revascularization. Secondary endpoints included individual MACE components (total death, cardiac death, MI, revascularization, target lesion revascularization [TLR], target vessel revascularization [TVR], and non-TVR), stent thrombosis, and heart failure (HF)-related rehospitalization. All deaths were considered cardiac unless a definitive non-cardiac cause was established. Recurrent MI was defined as new pathological Q waves or re-elevation of cardiac biomarkers (troponin or creatine kinase–MB) with ischemic symptoms or electrocardiographic changes (11). Revascularization events were adjudicated based on angiographic documentation of ischemia-driven PCI or coronary artery bypass grafting (CABG). TLR was defined as revascularization of the treated lesion (repeat PCI or CABG). TVR was defined as revascularization of the treated vessel.

### 2.3 Follow-up protocol

Clinical follow-up was scheduled at 1, 6, 12, and 36 months post-discharge through outpatient clinic visits, telephone interviews, medical record review, and at any clinical event. All clinical events were identified by treating physicians, verified through medical record review, and confirmed by the principal investigator at each participating hospital.

### 2.4 PCI procedures

PCI procedures followed contemporary clinical practice guidelines using standard techniques (12). Conventional PCI guidewires, balloons, thrombus aspiration devices, and adjunctive devices were used appropriately, with plaque modification techniques (non-compliant balloons, scoring or cutting balloons) applied selectively based on angiographic and lesion-specific characteristics. All PCI procedures used DES following adequate lesion preparation. Selection DES type and use of adjunctive intracoronary imaging modalities (intravascular ultrasound or optical coherence tomography) were at operator discretion. Procedural success was defined as residual angiographic diameter stenosis <30% with TIMI grade 3 flow restoration (13). All patients received guideline-recommended antiplatelet therapy before PCI, including loading doses of aspirin (300 mg) and a P2Y12 inhibitor (clopidogrel 600 mg, prasugrel 60 mg, or ticagrelor 180 mg). Following PCI, dual antiplatelet therapy (DAPT) with aspirin and a P2Y12 inhibitor was prescribed for ≥12 months unless contraindicated. DAPT duration and antiplatelet agent choice after transitioning to single antiplatelet therapy followed current guidelines and physician's discretion (13). Statin therapy was mandatory, and other concomitant cardiovascular beneficial medications, including renin–angiotensin system inhibitors (RASi) and additional guideline-directed medical therapies, were prescribed at physician's discretion.

### 2.5 Statistical analyses

Patients were categorized into treatment groups based on the initial medication prescribed at hospital discharge, and all clinical outcome analyses were conducted according to a pseudo-intention-to-treat (pseudo-ITT) principle. Continuous variables are expressed as mean ± standard deviation or median with interquartile ranges; categorical variables as counts with percentages. Between-group comparisons used Student's *t*-test or Mann–Whitney *U*-test for continuous variables and *χ*^2^ or Fisher's exact test for categorical variables. To minimize treatment-selection bias and confounding, inverse probability of treatment weighting (IPTW) based on propensity scores was applied. Propensity scores were estimated using logistic regression including all relevant baseline, angiographic, and discharge medication variables. Covariate balance after weighting was assessed using standardized mean differences (SMD <0.10 considered well balanced). Clinical outcomes were evaluated using Cox proportional hazards regression to calculate hazard ratios (HRs) and 95% confidence intervals (CIs). Variables with residual imbalance after IPTW adjustment (e.g., triglyceride levels), together with clinically relevant baseline characteristics, were additionally incorporated as covariates in the multivariable Cox proportional hazards regression models. The Kaplan–Meier method plotted event-free survival curves, with differences tested by log-rank method. Subgroup analyses were performed for prespecified variables, including sex, age (<65 vs. ≥65 years), LVEF, Killip class, hypertension, diabetes mellitus, and multivessel disease status. Interaction terms were tested to explore treatment effect heterogeneity. A restricted cubic spline (RCS) analysis was performed to assess the continuous relationship between baseline LVEF and the hazard ratio for 3-year MACE associated with nebivolol vs. carvedilol. All analyses were two-tailed, with *p* < 0.05 considered statistically significant. Statistical computations used SPSS version 20.0 (SPSS Inc., Chicago, IL) and R version 4.2.2 (R Foundation for Statistical Computing, Vienna, Austria).

## 3 Results

A total of 6,752 in-hospital survivors with STEMI who underwent successful PCI using second-generation DES and were prescribed vasodilative *β*-blockers were included in the analysis. Of these, 4,725 patients received carvedilol and 2,027 received nebivolol at discharge. After IPTW adjustment, 6,642 patients in the nebivolol group and 6,749 patients in the carvedilol group were included in the weighted analysis (Figure 1).

### 3.1 Baseline clinical and laboratory characteristics

Baseline clinical and laboratory characteristics are presented in Table 1. In the crude population, patients treated with nebivolol had a higher LVEF than those treated with carvedilol (51.5 ± 10.1% vs. 49.9 ± 9.8%, *p* < 0.001). Dyslipidemia and a history of smoking were more prevalent in the carvedilol group (13.3% vs*.* 11.1%, *p* = 0.016; 63.5% vs*.* 60.1%, *p* = 0.009), including current smoking (46.6% vs. 43.4%, *p* = 0.015). Serum creatinine levels were slightly higher in the carvedilol group (1.03 ± 0.85 mg/dL vs. 1.00 ± 0.53 mg/dL, *p* = 0.027), whereas triglyceride levels were higher in the nebivolol group (156 ± 139 mg/dL vs. 147 ± 127 mg/dL, *p* = 0.021). After IPTW adjustment, the absolute standardized mean differences (SMDs) for all covariates were <0.10, indicating excellent balance between the two groups. Although triglyceride levels remained statistically different because of the large sample size (156 ± 138 mg/dL vs. 147 ± 127 mg/dL, *p* < 0.001), the SMD remained low at 0.06, supporting adequate clinical balance.

**Table 1:** Baseline clinical characteristics and laboratory findings.

Variables, *n* (%)	Crude population				IPTW population			
	Nebivolol (*n* = 2,027)	Carvedilol (*n* = 4,725)	*p*-value	SMD	Nebivolol (*n* = 6,642)	Carvedilol (*n* = 6,749)	*p*-value	SMD
Sex, male	1,662 (82.0)	3,850 (81.5)	0.619	−0.06	5,437 (81.9)	5,512 (81.7)	0.780	−0.02
Age, yrs	62.2 ± 12.5	61.6 ± 12.3	0.052	0.05	62 ± 12.7	61.8 ± 12.3	0.361	0.02
Body mass index, kg/m^2^	24.3 ± 3.2	24.4 ± 3.2	0.427	−0.02	24.4 ± 3.3	24.4 ± 3.2	0.426	0.01
LV ejection fraction, %	51.5 ± 10.1	49.9 ± 9.8	<0.001	0.16	50.1 ± 10.2	50.3 ± 9.8	0.266	−0.02
Killip class (III/IV)								
Class III	83 (4.1)	158 (3.3)	0.127	−0.39	229 (3.4)	238 (3.5)	0.803	0.04
Class IV	115 (5.7)	292 (6.2)	0.423	0.21	366 (5.5)	401 (5.9)	0.283	0.18
Risk of Patients								
Hypertension	946 (46.7)	2,148 (45.5)	0.361	−0.18	3,081 (46.4)	3,092 (45.8)	0.511	−0.08
Diabetes mellitus	490 (24.2)	1,113 (23.6)	0.584	−0.13	1,632 (24.6)	1,584 (23.5)	0.136	−0.22
Dyslipidemia	226 (11.1)	627 (13.3)	0.016	0.61	830 (12.5)	854 (12.7)	0.783	0.04
Prior myocardial infarction	73 (3.6)	192 (4.1)	0.370	0.24	244 (3.7)	263 (3.9)	0.498	0.11
Prior Stroke	104 (5.1)	221 (4.7)	0.425	−0.20	347 (5.2)	320 (4.7)	0.200	−0.22
Prior Heart Failure	10 (0.5)	25 (0.5)	0.851	0.05	44 (0.7)	35 (0.5)	0.278	−0.19
Smoking history	1,218 (60.1)	2,999 (63.5)	0.009	0.43	4,154 (62.5)	4,221 (62.5)	0.999	0.00
Current smoker	879 (43.4)	2,201 (46.6)	0.015	0.48	3,008 (45.3)	3,083 (45.7)	0.648	0.06
Laboratory findings								
Glucose (mg/dL)	173 ± 71	172 ± 74	0.891	0.00	172 ± 70	172 ± 75	0.875	0.00
X`HbA1c (%)	6.4 ± 1.4	6.4 ± 1.5	0.270	−0.03	6.4 ± 1.4	6.4 ± 1.5	0.977	0.00
Creatinine (mg/dL)	1.00 ± 0.53	1.03 ± 0.85	0.027	−0.05	1.03 ± 0.68	1.03 ± 0.79	0.492	0.01
Total cholesterol (mg/dL)	183 ± 46	183 ± 44	0.974	0.00	184 ± 48	183 ± 44	0.120	0.03
Triglyceride (mg/dL)	156 ± 139	147 ± 127	0.021	0.07	156 ± 138	147 ± 127	<0.001	0.06
HDL-cholesterol (mg/dL)	44 ± 12	44 ± 12	0.900	0.00	44 ± 11	44 ± 12	0.427	−0.01
LDL-cholesterol (mg/dL)	110 ± 39	111 ± 40	0.593	−0.01	111 ± 39	110 ± 40	0.669	0.01
BNP (pg/mL)	154 ± 370	163 ± 455	0.735	0.05	167 ± 430	157 ± 436	0.573	−0.02

IPTW, inverse probability of treatment weighting; SMD, standardized mean difference; LVEF, left-ventricular ejection fraction; HbA1c, hemoglobin A1c; HDL, high-density lipoprotein; LDL, low-density lipoprotein; BNP, B-type Natriuretic Peptide.

### 3.2 Angiographic, procedural characteristics and medications

Angiographic and procedural characteristics and discharge medications are summarized in Table 2. In the crude population, multivessel disease was more common in the carvedilol group (43.3% vs. 39.4%, *p* = 0.003), with a greater number of diseased vessels (1.59 ± 0.74 vs. 1.51 ± 0.70, *p* < 0.001). Sirolimus- and everolimus-eluting stents were used more frequently in the nebivolol group (21.9% vs. 18.3%, *p* = 0.001; 50.1% vs. 46.4%, *p* = 0.005), whereas zotarolimus- and biolimus-eluting stents were more commonly used in the carvedilol group (21.9% vs. 19.2%, *p* = 0.015; 11.9% vs. 6.0%, *p* < 0.001).

**Table 2:** Angiographic and procedural characteristics and discharge medications.

Variables, *n* (%)	Crude population				IPTW population			
	Nebivolol (*n* = 2,027)	Carvedilol (*n* = 4,725)	*p*-value	SMD	Nebivolol (*n* = 6,642)	Carvedilol (*n* = 6,749)	*p*-value	SMD
Angiographic and procedural characteristics								
Stent types								
Sirolimus-eluting	443 (21.9)	866 (18.3)	0.001	−0.79	1,313 (19.8)	1,303 (19.3)	0.503	−0.10
Zotarolimus-eluting	390 (19.2)	1,033 (21.9)	0.015	0.58	1,360 (20.5)	1,418 (21.0)	0.445	0.12
Everolimus-eluting	1,016 (50.1)	2,193 (46.4)	0.005	−0.54	3,203 (48.2)	3,229 (47.8)	0.660	−0.05
BA9-eluting	122 (6.0)	564 (11.9)	<0.001	1.98	681 (10.3)	688 (10.2)	0.911	−0.02
Novolimus-eluting	37 (1.8)	69 (1.5)	0.269	−0.28	94 (1.4)	102 (1.5)	0.643	0.08
Multi-vessel isease	799 (39.4)	2,046 (43.3)	0.003	0.61	2,804 (42.2)	2,848 (42.2)	0.984	0.00
No. of diseased vessel, N	1.51 ± 0.7	1.59 ± 0.74	<0.001	−0.10	1.57 ± 0.73	1.57 ± 0.73	0.816	0.00
Infarct-related artery								
Left main	59 (2.9)	121 (2.6)	0.413	−0.21	202 (3.0)	175 (2.6)	0.117	−0.27
LAD	1,233 (60.8)	2,922 (61.8)	0.433	0.13	4,209 (63.4)	4,130 (61.2)	0.009	−0.28
LCx	356 (17.6)	844 (17.9)	0.768	0.07	1,253 (18.9)	1,189 (17.6)	0.062	−0.29
RCA	874 (43.1)	1,955 (41.4)	0.184	−0.27	2,748 (41.4)	2,825 (41.9)	0.569	0.08
Discharge medications								
Aspirin	2,015 (99.4)	4,696 (99.4)	0.916	0.00	6,593 (99.3)	6,707 (99.4)	0.416	0.01
Clopidogrel	737 (36.4)	2,278 (48.2)	<0.001	1.83	2,970 (44.7)	3,013 (44.6)	0.933	−0.01
Cilostazol	59 (2.9)	272 (5.8)	<0.001	1.37	347 (5.2)	333 (4.9)	0.444	−0.13
Ticagrelor	1,100 (54.3)	1,971 (41.7)	<0.001	−1.82	3,001 (45.2)	3,068 (45.5)	0.748	0.04
Prasugrel	189 (9.3)	468 (9.9)	0.461	0.19	659 (9.9)	660 (9.8)	0.782	−0.05
Ca-channel blockers	54 (2.7)	128 (2.7)	0.917	0.03	185 (2.8)	182 (2.7)	0.755	−0.05
RAS inhibitors	1,721 (84.9)	3,696 (78.2)	<0.001	−0.74	5,368 (80.8)	5,396 (80.0)	0.213	−0.10
ARBs	681 (33.6)	1,335 (28.3)	<0.001	−0.96	2,068 (31.1)	2,042 (30.3)	0.270	−0.16
ACE inhibitors	1,040 (51.3)	2,383 (50.4)	0.511	−0.12	3,301 (49.7)	3,388 (50.2)	0.556	0.07
Statin	1,974 (97.4)	4,563 (96.6)	0.081	−0.08	6,430 (96.8)	6,531 (96.8)	0.900	0.00

IPTW, inverse probability of treatment weighting; SMD, standardized mean difference; BA9, biolimus A9; LAD, left anterior descending artery; LCx, left circumflex artery; RCA, right coronary artery; RASi, renin–angiotensin system inhibitor; ARB, angiotensin receptor blocker; ACEi, angiotensin-converting enzyme inhibitor.

Regarding discharge medications, clopidogrel and cilostazol were prescribed more frequently in the carvedilol group (48.2% vs. 36.4%, *p* < 0.001; 5.8% vs. 2.9%, *p* < 0.001). In contrast, ticagrelor and RASi were more frequently prescribed in the nebivolol group (54.3% vs. 41.7%, *p* < 0.001; 84.9% vs. 78.2%, *p* < 0.001), including angiotensin receptor blocker (ARB; 33.6% vs. 28.3%, *p* < 0.001). After IPTW adjustment, angiographic characteristics and discharge medications were well balanced, except for infarct-related artery distribution, with left anterior descending artery involvement remaining slightly more frequent in the nebivolol group (63.4% vs. 61.2%, *p* = 0.009).

### 3.3 Long-Term clinical outcomes

**Figure 2:**
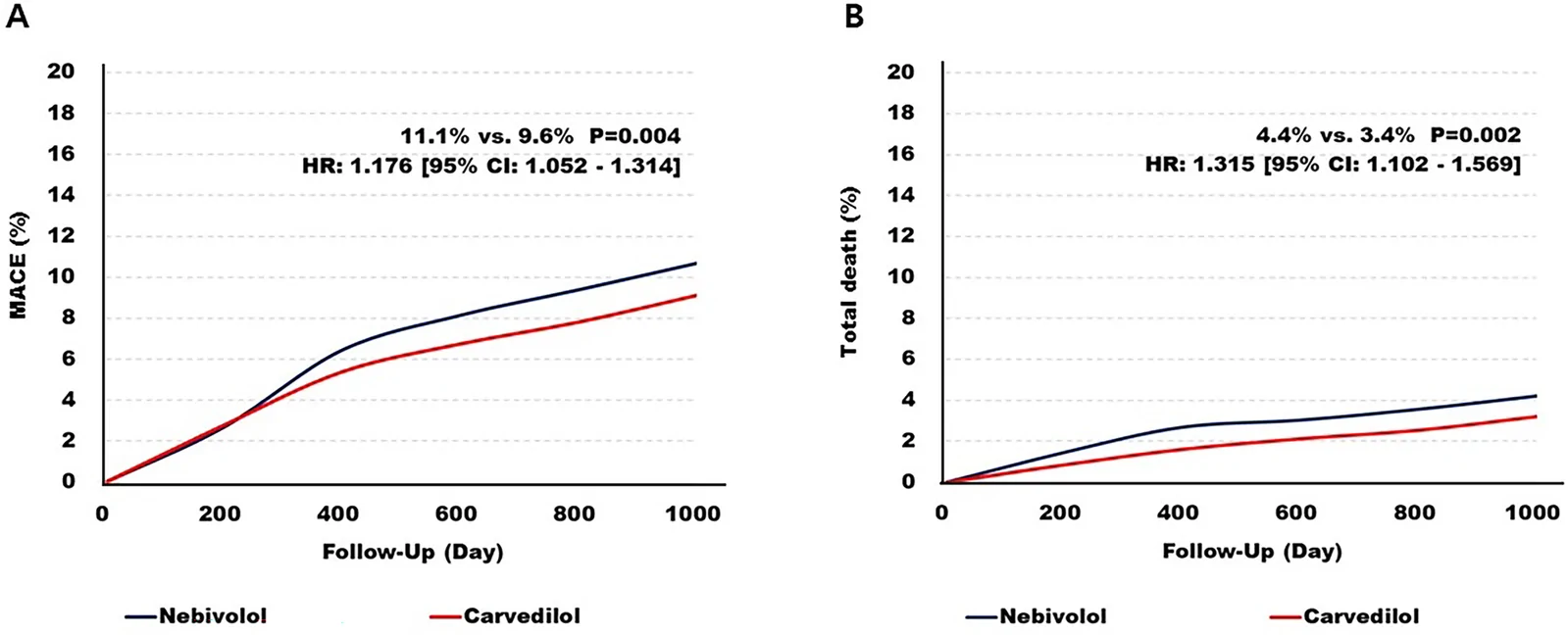
Kaplan–meier curves for **(A)** major adverse cardiovascular events (MACE) and **(B)** all-cause death in the inverse probability of treatment–weighted population. At 3 years, the cumulative incidence of MACE was higher in the nebivolol group than in the carvedilol group (11.1% vs. 9.6%, log-rank *p* = 0.004). The cumulative incidence of all-cause death at 3 years was also higher in the nebivolol group (4.4% vs. 3.4%, log-rank *p* = 0.002). CI, confidence interval; CTO, chronic total occlusion; HF, heart failure; HR, hazard ratio; IPTW, inverse probability weighting; MACE, major adverse cardiovascular events (defined as a composite of total death, myocardial infarction, and revascularization); MACCE, major adverse cardiovascular events; MT, medical therapy; PCI, percutaneous coronary intervention; STEMI, ST-segment elevation myocardial infarction.

Subsequently, clinical outcomes at 1 year and 3 years were compared between the nebivolol and carvedilol groups using Cox proportional hazards models before and after IPTW adjustment (Table 3). Cumulative event rates were evaluated using Kaplan–Meier analyses (Figure 2).

**Table 3:** Three-Year clinical outcomes before and after IPTW adjustment.

Variables, *n* (%)	Crude population					IPTW population				
	Nebivolol (*n* = 2,027)	Carvedilol (*n* = 4,725)	*p*-value	HR (95% CI)	*p*-value	Nebivolol (*n* = 6,642)	Carvedilol (*n* = 6,749)	*p*-value	HR (95% CI)	*p*-value
1-year outcomes										
Total death	34 (1.7)	73 (1.5)	0.690	1.087 (0.721–1.638)	0.672	150 (2.3)	104 (1.5)	0.002	1.476 (1.146–1.899)	0.003
Cardiac death	16 (0.8)	39 (0.8)	0.880	0.955 (0.532–1.714)	>0.999	62 (0.9)	56 (0.8)	0.521	1.125 (0.783–1.618)	0.579
Myocardial infarction	15 (0.7)	34 (0.7)	0.928	1.028 (0.559–1.892)	>0.999	53 (0.8)	47 (0.7)	0.495	1.146 (0.773–1.701)	0.547
STEMI	8 (0.4)	17 (0.4)	0.829	1.097 (0.472–2.546)	0.829	31 (0.5)	23 (0.3)	0.250	1.371 (0.798–2.354)	0.277
Revascularization	59 (2.9)	156 (3.3)	0.402	0.878 (0.647–1.190)	0.450	190 (2.9)	216 (3.2)	0.251	0.890 (0.730–1.085)	0.268
Target lesion: TLR	16 (0.8)	49 (1.0)	0.339	0.759 (0.430–1.338)	0.415	52 (0.8)	64 (0.9)	0.302	0.824 (0.570–1.190)	0.307
Target vessel: TVR	23 (1.1)	76 (1.6)	0.138	0.702 (0.439–1.122)	0.152	73 (1.1)	105 (1.6)	0.021	0.703 (0.520–0.949)	0.024
Non-TVR	28 (1.4)	79 (1.7)	0.381	0.823 (0.533–1.271)	0.457	97 (1.5)	110 (1.6)	0.426	0.894 (0.679–1.177)	0.441
Stroke	20 (1.0)	45 (1.0)	0.895	1.036 (0.610–1.759)	0.892	74 (1.1)	64 (0.9)	0.343	1.176 (0.840–1.647)	0.348
Stent thrombosis	11 (0.5)	18 (0.4)	0.352	1.426 (0.672–3.026)	0.416	42 (0.6)	27 (0.4)	0.061	1.584 (0.975–2.572)	0.070
HF related rehospitalization	32 (1.6)	85 (1.8)	0.525	0.875 (0.581–1.319)	0.611	123 (1.9)	117 (1.7)	0.606	1.069 (0.828–1.380)	0.649
MACE	97 (4.8)	242 (5.1)	0.562	0.931 (0.731–1.185)	0.585	354 (5.3)	339 (5.0)	0.424	1.064 (0.913–1.240)	0.435
3-year outcomes										
Total death	73 (3.6)	160 (3.4)	0.657	1.065 (0.804–1.412)	0.663	292 (4.4)	228 (3.4)	0.002	1.315 (1.102–1.569)	0.002
Cardiac death	34 (1.7)	81 (1.7)	0.914	0.978 (0.653–1.464)	>0.999	136 (2.0)	117 (1.7)	0.182	1.184 (0.923–1.520)	0.183
Myocardial infarction	35 (1.7)	83 (1.8)	0.931	0.982 (0.659–1.463)	>0.999	139 (2.1)	115 (1.7)	0.099	1.232 (0.960–1.582)	0.100
STEMI	14 (0.7)	34 (0.7)	0.897	0.959 (0.513–1.791)	>0.999	52 (0.8)	46 (0.7)	0.492	1.149 (0.772–1.712)	0.543
Revascularization	137 (6.8)	298 (6.3)	0.488	1.076 (0.873–1.327)	0.483	467 (7.0)	416 (6.2)	0.043	1.151 (1.004–1.319)	0.043
Target lesion: TLR	44 (2.2)	95 (2.0)	0.671	1.081 (0.753–1.551)	0.708	155 (2.3)	131 (1.9)	0.117	1.206 (0.953–1.526)	0.120
Target vessel: TVR	62 (3.1)	151 (3.2)	0.768	0.955 (0.707–1.290)	0.820	214 (3.2)	212 (3.1)	0.790	1.026 (0.846–1.245)	0.806
Non-TVR	60 (3.0)	143 (3.0)	0.886	0.977 (0.720–1.328)	0.938	220 (3.3)	197 (2.9)	0.188	1.140 (0.937–1.385)	0.196
Stroke	34 (1.7)	84 (1.8)	0.773	0.942 (0.630–1.408)	0.840	126 (1.9)	118 (1.7)	0.520	1.086 (0.843–1.400)	0.561
Stent thrombosis	16 (0.8)	33 (0.7)	0.687	1.131 (0.621–2.059)	0.754	63 (0.9)	49 (0.7)	0.158	1.309 (0.900–1.904)	0.184
HF related rehospitalization	50 (2.5)	129 (2.7)	0.537	0.901 (0.647–1.254)	0.564	196 (3.0)	185 (2.7)	0.465	1.078 (0.879–1.322)	0.467
MACE	208 (10.3)	463 (9.8)	0.560	1.052 (0.885–1.250)	0.564	740 (11.1)	650 (9.6)	0.004	1.176 (1.052–1.314)	0.005

IPTW, inverse probability of treatment weighting; HR, hazard ratio; CI, confidence interval; STEMI, ST-elevation myocardial infarction; TLR, target lesion revascularization; TVR, target vessel revascularization; HF, heart failure; MACE, major adverse cardiovascular events.

In the crude population, the incidence of MACE did not differ significantly between groups at either 1 year or 3 years. Likewise, no significant between-group differences were observed in secondary outcomes, including total death, cardiac death, myocardial infarction, revascularization, stent thrombosis, and HF-related rehospitalization.

After IPTW adjustment, the 1-year incidence of MACE remained similar between groups (5.3% vs. 5.0%, *p* = 0.424; HR: 1.06; 95% CI: 0.91–1.24; *p* = 0.435). At 3 years, however, the nebivolol group showed a significantly higher MACE risk than the carvedilol group (11.1% vs. 9.6%, *p* = 0.004), a finding confirmed by IPTW-adjusted Cox analysis (HR: 1.18; 95% CI: 1.05–1.31; *p* = 0.005) and Kaplan–Meier analysis (Figure 2A).

Total death occurred more frequently in the nebivolol group after IPTW adjustment at both 1 year (2.3% vs. 1.5%, *p* = 0.002; HR: 1.48; 95% CI: 1.15–1.90; *p* = 0.003) and 3 years (4.4% vs. 3.4%, *p* = 0.002; HR: 1.32; 95% CI: 1.10–1.57; *p* = 0.002), with early and persistent separation of the Kaplan–Meier curves favouring carvedilol (Figure 2B).

Revascularization-related outcomes did not differ between groups in the crude population. After IPTW adjustment, revascularization at 3 years was more frequent in the nebivolol group (7.0% vs. 6.2%, *p* = 0.043; HR: 1.15; 95% CI: 1.00–1.32; *p* = 0.043), whereas TVR at 1 year occurred less frequently (1.1% vs. 1.6%; HR: 0.70; 95% CI: 0.52–0.95; *p* = 0.024). No other secondary outcomes differed significantly after IPTW adjustment.

**Figure 3:**
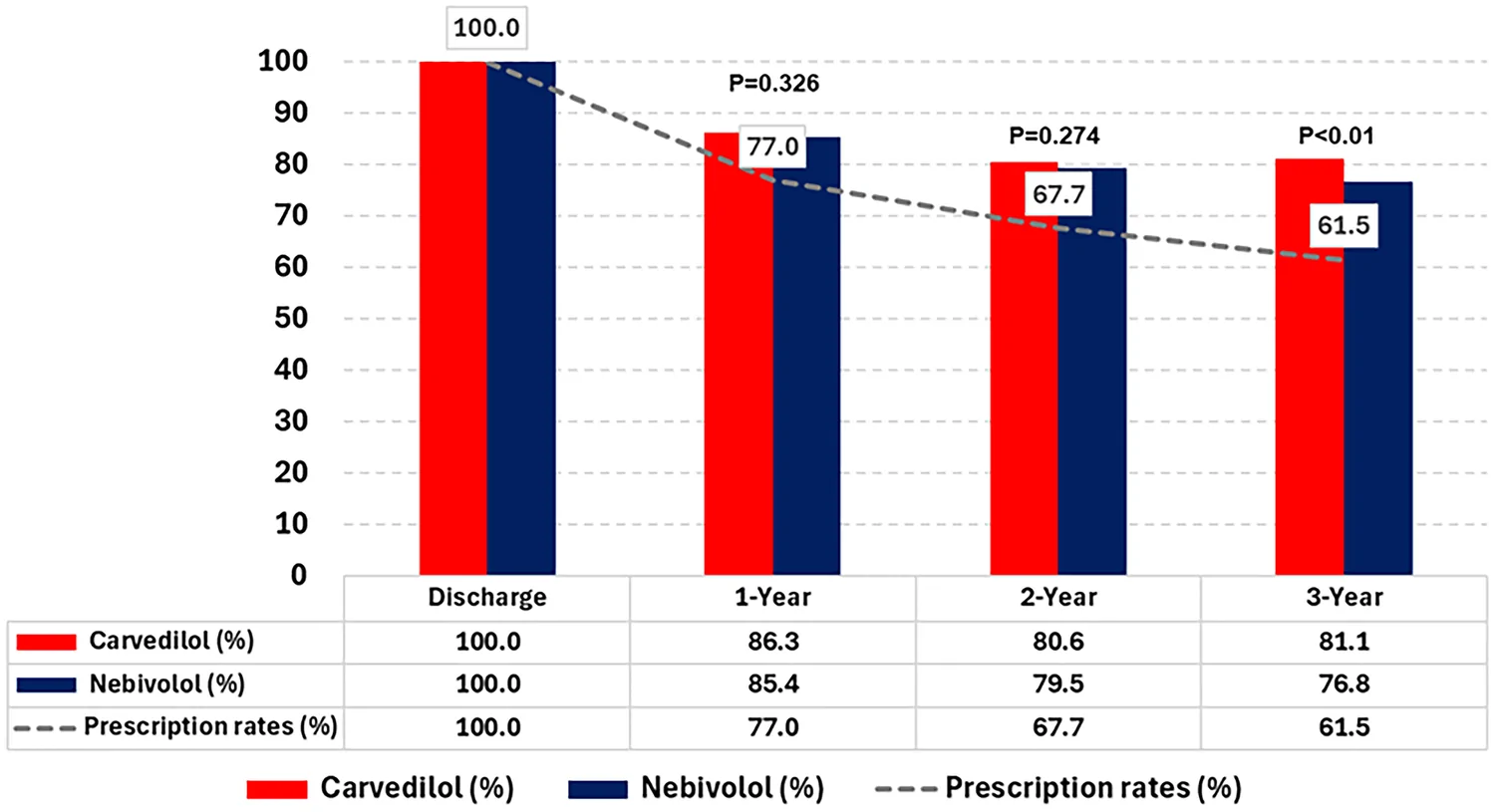
Comparison of prescription adherence rates between nebivolol and carvedilol during the 3-year follow-up.

Alongside these clinical endpoints, longitudinal changes in prescription adherence and hemodynamic parameters were evaluated. During the 3-year follow-up, the prescription adherence rate was comparable between the two groups up to 2 years; however, the carvedilol group maintained a significantly higher adherence rate at 3 years compared to the nebivolol group (81.1% vs. 76.8%, *p* < 0.01) (Figure 3). Furthermore, while both treatments improved echocardiographic and hemodynamic profiles from baseline to 36 months (Table 4), the magnitude of LVEF improvement was significantly greater in the carvedilol group compared to the nebivolol group (*Δ* −4.2 ± 8.8% vs. −2.8 ± 9.9%, *p* = 0.005). Conversely, the nebivolol group exhibited a more pronounced reduction in heart rate (*Δ* 4.6 ± 21.2 vs. 2.6 ± 20.5 bpm, *p* < 0.001), with both groups showing comparable reductions in systolic and diastolic blood pressures.

**Table 4:** Changes in hemodynamic and echocardiographic parameters from baseline to 3-year follow-up.

		Crude population				IPTW population			
		Nebivolol	Carvedilol	*p*-value	SMD	Nebivolol	Carvedilol	*p*-value	SMD
		(*n* = 2,027)	(*n* = 4,725)			(*n* = 6,642)	(*n* = 6,749)		
LVEF	Initial	51.5 ± 10.1	49.9 ± 9.8	<0.001	0.16	50.1 ± 10.2	50.3 ± 9.8	0.266	−0.02
	36M	53.6 ± 11.2	53.0 ± 10.6	0.515	0.05	52.1 ± 11.3	53.5 ± 10.6	0.015	−0.13
	*Δ* (Initial−36M)	−2.5 ± 9.9	−4.2 ± 8.9	0.026	0.18	−2.8 ± 9.9	−4.2 ± 8.8	0.005	0.15
SBP	Initial	129.6 ± 28.4	131.3 ± 28.2	0.021	−0.06	131.4 ± 29.3	130.8 ± 28.2	0.265	0.02
	36M	126.2 ± 14.9	126.3 ± 15.2	0.921	0.00	126.2 ± 15.0	126.4 ± 15.1	0.622	−0.01
	*Δ* (Initial−36M)	2.5 ± 30.3	6.5 ± 30.3	0.001	−0.13	4.5 ± 30.4	5.5 ± 30.1	0.194	−0.03
DBP	Initial	79.0 ± 17.7	80.3 ± 17.7	0.004	−0.08	80.0 ± 18.2	79.9 ± 17.7	0.587	0.01
	36M	73.3 ± 10.9	73.8 ± 11.3	0.262	−0.04	73.3 ± 11.0	73.8 ± 11.3	0.092	−0.04
	*Δ* (Initial−36M)	5.6 ± 19.9	7.6 ± 19.2	0.009	−0.10	6.9 ± 20.2	7.0 ± 19.0	0.791	−0.01
HR	Initial	77.9 ± 19.3	77.5 ± 18.9	0.423	0.02	78.5 ± 19.6	77.8 ± 18.9	0.052	0.04
	36M	73.9 ± 11.5	74.8 ± 11.6	0.049	−0.08	74.0 ± 11.4	74.9 ± 11.5	0.003	−0.08
	*Δ* (Initial−36M)	4.0 ± 20.9	2.8 ± 20.6	0.125	0.06	4.6 ± 21.2	2.6 ± 20.5	<0.001	0.10

IPTW, inverse probability of treatment weighting; SMD, standardized mean difference; LVEF, left ventricular ejection fraction; M, months; SBP, systolic blood pressure; DBP, diastolic blood pressure; HR, heart rate.

### 3.4 Subgroup analyses

**Figure 4:**
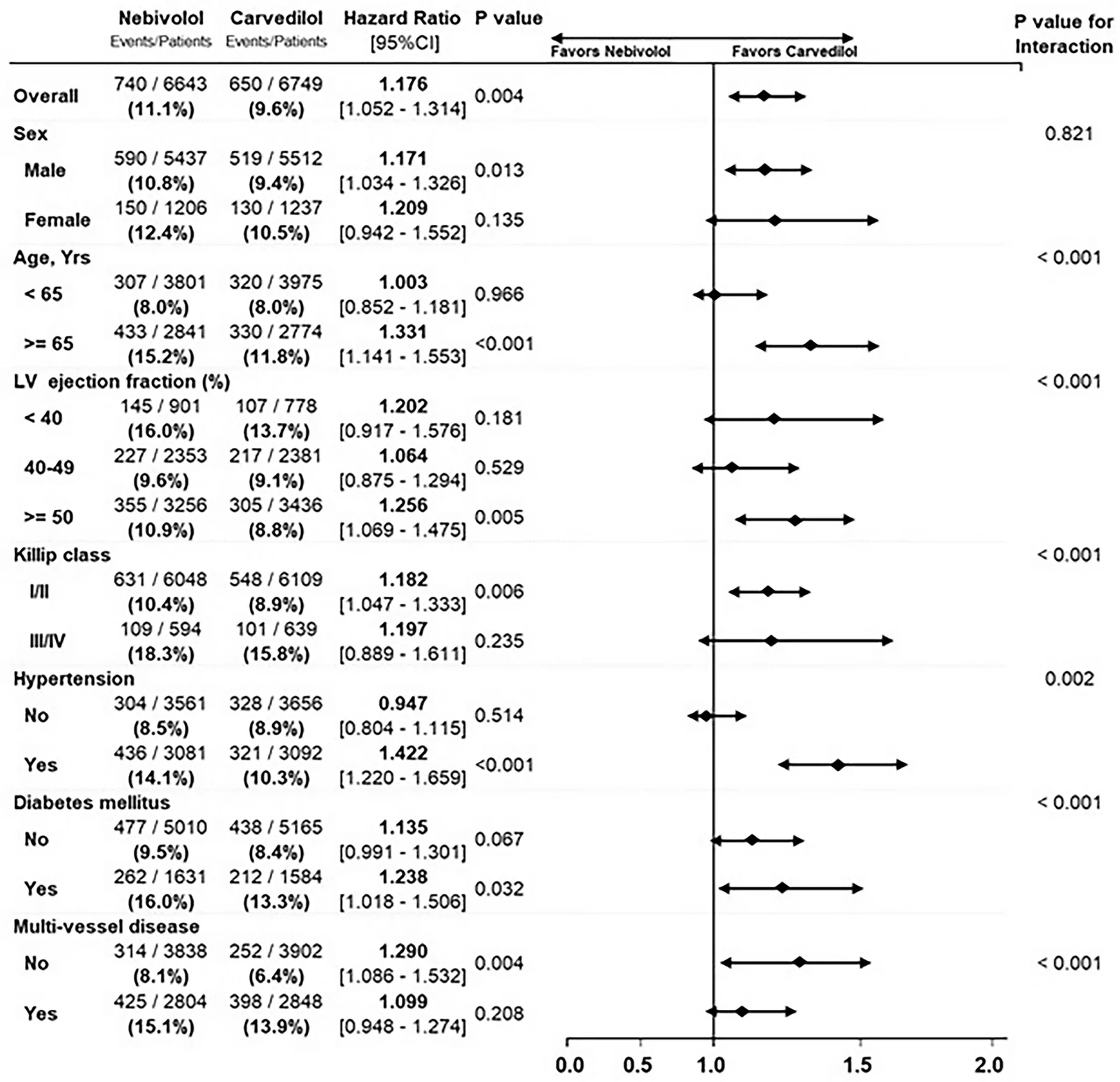
Subgroup analysis of major adverse cardiac events (MACE) after inverse probability of treatment weighting. IPTW, inverse probability of treatment weighting; MACE, major adverse cardiac events; HR, hazard ratio; CI, confidence interval; LV, left ventricular; LVEF, left ventricular ejection fraction.

Prespecified subgroup analyses for the primary outcome are shown in Figure 4. In the IPTW-adjusted population, significant treatment-by-subgroup interactions were observed for age, LVEF, Killip class, hypertension, diabetes mellitus, multivessel disease, and mode of presentation (all *p* for interaction <0.01). No significant interaction was observed for sex (*p* for interaction = 0.821).

**Figure 5:**
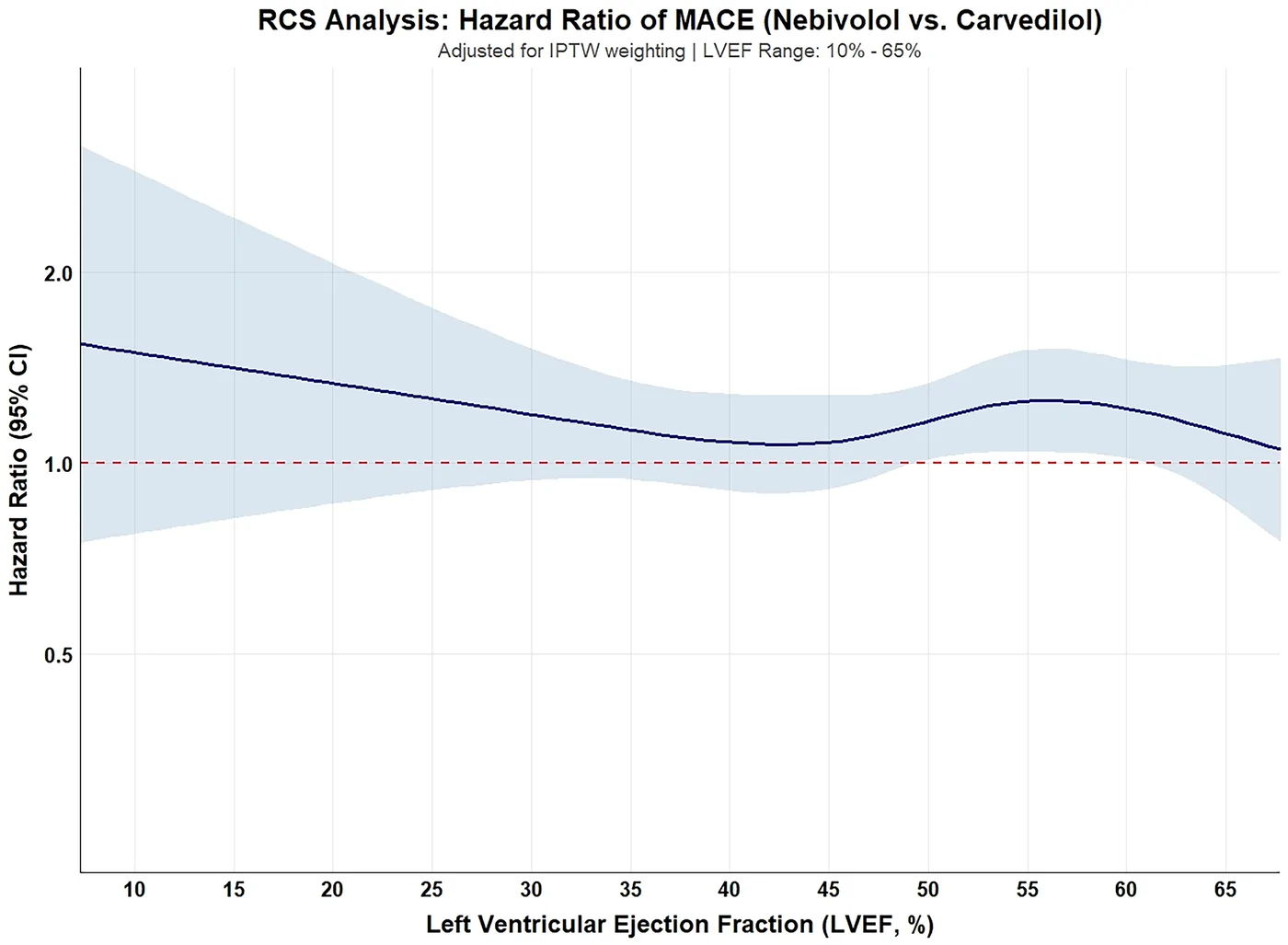
Restricted cubic spline (RCS) curve for the hazard ratio of 3-year major adverse cardiovascular events (MACE) according to baseline left ventricular ejection fraction (LVEF). The solid line and shaded area represent the hazard ratio for nebivolol versus carvedilol and its 95% confidence interval, respectively. The red dashed line indicates a hazard ratio of 1.0. The hazard ratio remained consistently above 1.0 across the full range of baseline LVEF values, indicating a consistently higher risk of MACE with nebivolol compared with carvedilol. CI, confidence interval; LVEF, left ventricular ejection fraction; MACE, major adverse cardiovascular events; RCS, restricted cubic spline.

Among subgroups with significant interaction, the magnitude of risk varied. Higher HRs for MACE were observed in patients aged ≥65 years (HR: 1.331; 95% CI: 1.141–1.553), those with LVEF ≥50% (HR: 1.256; 95% CI: 1.069–1.475), patients with Killip class I or II (HR: 1.182; 95% CI: 1.047–1.333), patients with hypertension (HR: 1.422; 95% CI: 1.220–1.659), patients with diabetes mellitus (HR: 1.238; 95% CI: 1.018–1.506), and patients without multivessel disease (HR: 1.290; 95% CI: 1.086–1.532). Furthermore, to evaluate the treatment effect across the full range of baseline LVEF, an RCS analysis was performed. As shown in Figure 5, the hazard ratio for 3-year MACE (nebivolol vs. carvedilol) remained consistently above 1.0 across baseline LVEF values, supporting the consistency of the treatment effect across different clinical strata.

## 4 Discussion

In this large, contemporary registry of patients with STEMI undergoing primary PCI with second-generation DES, we present a head-to-head comparison of long-term clinical outcomes between two vasodilating *β*-blockers, carvedilol and nebivolol, before and after IPTW adjustment. Principal findings include: 1) After IPTW adjustment, carvedilol demonstrated significantly lower 3-year MACE incidence vs. nebivolol. 2) This difference was primarily driven by reduced incidence of total death and revascularization with carvedilol, showing early and sustained survival curve separation. 3) Although individual secondary endpoints did not uniformly achieve statistical significance, most ischemic and cardiac-related MACE components numerically favoured carvedilol, indicating a consistent directional benefit beyond isolated mortality reduction. 4) Carvedilol's relative benefit remained consistent across clinically relevant subgroups, with particularly robust associations in older patients, those with preserved LVEF, lower Killip class, and cardiometabolic comorbidities.

*β*-blockers represent a cornerstone of guideline-directed medical therapy for patients with HF and ischemic heart disease. Current international guidelines recommend long-term *β*-blocker therapy as Class I in patients with reduced LVEF and Class IIa in selected patients post-MI (1, 2). However, supporting evidence derives largely from an era predating widespread routine revascularization and contemporary stent technology. Data specifically addressing outcomes in patients receiving primary PCI with second-generation DES therefore remain sparse.

Concurrent with revascularization and secondary prevention advances, vasodilating *β*-blockers have garnered increasing attention in contemporary MI management. Multiple observational and mechanistic studies suggest vasodilating *β*-blockers offer superior clinical profiles vs. conventional *β*-blockers regarding endothelial function, metabolic effects, and long-term outcomes, though primarily from heterogeneous populations (7, 8, 14). Despite these observations, direct comparative data between specific vasodilating *β*-blockers in PCI-treated populations with MI remain scarce. Head-to-head comparisons between carvedilol and nebivolol, both established vasodilating *β*-blockers, in patients with STEMI receiving primary PCI with second-generation DES are virtually nonexistent.

To address this gap, we evaluated long-term outcomes associated with carvedilol vs. nebivolol in a large contemporary cohort of patients with STEMI treated with primary PCI using second-generation DES. Carvedilol demonstrated better long-term clinical outcomes, including reduced MACE and total death and revascularization compared with nebivolol. To our knowledge, this analysis represents among the first direct comparisons of these agents in this clinical context. Özaydın et al. previously investigated patients with MI and LV systolic dysfunction, reporting nebivolol superiority over metoprolol for the primary composite endpoint (*p* < 0.05); however, no significant difference between nebivolol and carvedilol (*p* > 0.05) (15). However, this earlier study included heterogeneous populations managed without routine reperfusion and preceded contemporary primary PCI and second-generation DES adoption. Our study focuses exclusively on contemporary patients with STEMI uniformly receiving primary PCI with second-generation DES, providing agent-specific comparative evidence demonstrating differential long-term outcomes between carvedilol and nebivolol in the contemporary PCI era.

Although both are vasodilating *β*-blockers, carvedilol and nebivolol exhibit substantially different pharmacologic profiles, particularly regarding *β*1-selectivity and NO-mediated vasodilation (7). Nebivolol, a highly *β*1-selective agent, enhances endothelial NO bioavailability, thereby improving endothelial function and vascular reactivity (16). Carvedilol provides nonselective *β*-adrenergic blockade with *α*1-adrenergic antagonism, producing peripheral vasodilation through distinct mechanisms that reduce afterload and modulate vascular tone (17). However, in acute MI and coronary stent implantation settings, dysregulated NO signalling—including reduced bioavailability and endothelial NO synthase uncoupling—correlates with persistent endothelial dysfunction, inflammation, and adverse cardiovascular outcomes (17, 18). Stent implantation, even with DES, induces delayed endothelial healing and chronic endothelial dysfunction, wherein NO-dependent vasodilatory pathways may prove impaired or insufficient for offsetting vascular injury (19).

Under these conditions, carvedilol's vasodilatory effects—less dependent on NO signalling and supplemented by *α*1-adrenergic blockade and antioxidant properties—may interact more favorably with the pathophysiology of STEMI and DES implantation, potentially explaining the observed long-term cardiovascular outcome differences vs. nebivolol (20).

Beyond cardiovascular mechanisms, systemic and metabolic differences may contribute to divergent total mortality. Carvedilol demonstrates superior metabolic effects vs. conventional *β*-blockers, including enhanced insulin sensitivity and attenuated glycemic deterioration, as evidenced by randomized trials (21). Additionally, carvedilol's antioxidant and free radical scavenging properties may provide broader organ protection beyond myocardial benefits, potentially influencing noncardiac outcomes during extended follow-up (22). While hypothesis-generating within observational contexts, these mechanisms offer biologically plausible explanations for carvedilol's association with improved clinical outcomes including reduced total mortality.

Consistent with these mechanistic advantages, our echocardiographic and hemodynamic analyses revealed divergent profiles between the two agents. While detailed dose-titration data were unavailable in the registry, the magnitude of heart rate reduction was used as a surrogate marker of *β*1-blockade intensity. Both groups demonstrated substantial heart rate lowering, suggesting adequate pharmacological activity. Notably, nebivolol, a highly *β*1-selective agent, was associated with an even greater reduction in heart rate. Despite the greater reduction in heart rate observed with nebivolol, carvedilol was associated with a significantly larger improvement in LVEF at 3 years. Previous short-term studies in non-ischemic heart failure populations have reported comparable improvements in LVEF between the two agents (23). In contrast, our long-term data from a contemporary STEMI cohort suggest that carvedilol's broader adrenergic blockade (*β* and *α*1) may promote more favorable myocardial reverse remodeling. This structural and functional recovery may, in turn, contribute to the observed reduction in hard clinical outcomes, including mortality.

Furthermore, prescription adherence at 3 years was significantly higher in the carvedilol group. Although this greater long-term persistence may be associated with superior functional recovery (i.e., LVEF improvement), the precise reasons for treatment discontinuation—whether intolerance, non-compliance, or perceived clinical improvement—could not be determined from the registry. Accordingly, differential adherence may have partially confounded long-term clinical outcomes. Nevertheless, prescription rates remained comparable between the two groups for up to 2 years, whereas the Kaplan–Meier curves for MACE and all-cause mortality began to diverge much earlier. Taken together, these findings suggest that the observed outcome differences are more likely related to the intrinsic cardioprotective effects of carvedilol rather than being solely attributable to differential medication adherence.

Subgroup analyses revealed generally consistent associations between carvedilol and improved outcomes across clinically relevant strata. Notably, statistically significant interaction emerged among patients with preserved LVEF, wherein carvedilol demonstrated superior outcomes vs. nebivolol. This finding holds particular relevance as patients with preserved LVEF constitute a substantial proportion of contemporary populations with STEMI receiving PCI, yet agent-specific comparative data remain limited (24). Our results suggest carvedilol may confer superior outcomes vs. nebivolol in selected subgroups with preserved LV function, though these exploratory observations require prospective validation.

In contemporary patients with STEMI receiving primary PCI with second-generation DES, carvedilol demonstrated better long-term clinical outcomes vs. nebivolol. These findings indicate agent-specific differences among vasodilating *β*-blockers may carry clinical relevance in the modern reperfusion era, warranting prospective investigation.

### 4.1 Study limitations

Despite investigating a large, real-world cohort of patients with STEMI receiving contemporary primary PCI with second-generation DES, several limitations should be considered when interpreting the results. First, this study was specifically designed as a head-to-head comparison between carvedilol and nebivolol among patients already receiving beta-blocker therapy. Therefore, our findings should not be interpreted as evidence regarding the overall efficacy of beta-blocker therapy compared with non-use in patients with STEMI undergoing contemporary PCI. Given the ongoing debate regarding the benefit of beta-blockers in the modern reperfusion era, further dedicated studies including a comparator group of non-users are warranted. Second, the retrospective observational design precludes causal inference. Although IPTW balanced measured baseline characteristics, unmeasured confounding cannot be excluded. Third, nonrandomized treatment assignment may reflect physician judgment, institutional patterns, or uncaptured patient characteristics. Treatment-selection bias persists despite rigorous statistical adjustment. Fourth, although we evaluated longitudinal prescription adherence over the 3-year follow-up, detailed information on dose intensity, titration, specific timing, and reasons for medication switching or discontinuation was not available in the registry. Consequently, dose-response relationships could not be assessed, and time-updated sensitivity analyses incorporating censoring for treatment crossover or discontinuation were not feasible. Nevertheless, discharge prescription together with longitudinal adherence assessment represents a pragmatic and widely accepted surrogate for treatment strategy in large-scale real-world registries. Fifth, registry-based outcome ascertainment through medical record review risks misclassification or underreporting, particularly for nonfatal events. Standardized definitions and centralized data collection mitigated this limitation. Finally, the single-registry setting may limit generalizability to other populations, healthcare systems, or ethnic groups.

## 5 Conclusions

In this contemporary PCI-treated STEMI cohort, carvedilol was associated with better 3-year clinical outcomes including lower MACE, all-cause mortality and revascularization vs. nebivolol. These observations suggest that agent-specific differences among vasodilating *β*-blockers may be relevant in clinical practice; however, prospective studies are warranted to confirm these findings.

## Data Availability

The original contributions presented in the study are included in the article/Supplementary Material, further inquiries can be directed to the corresponding author/s.
